# tRNAviz: explore and visualize tRNA sequence features

**DOI:** 10.1093/nar/gkz438

**Published:** 2019-05-25

**Authors:** Brian Y Lin, Patricia P Chan, Todd M Lowe

**Affiliations:** Department of Biomolecular Engineering, University of California Santa Cruz, CA 95064, USA

## Abstract

Transfer RNAs (tRNAs) are ubiquitous across the tree of life. Although tRNA structure is highly conserved, there is still significant variation in sequence features between clades, isotypes and even isodecoders. This variation not only impacts translation, but as shown by a variety of recent studies, nontranslation-associated functions are also sensitive to small changes in tRNA sequence. Despite the rapidly growing number of sequenced genomes, there is a lack of tools for both small- and large-scale comparative genomics analysis of tRNA sequence features. Here, we have integrated over 150 000 tRNAs spanning all domains of life into tRNAviz, a web application for exploring and visualizing tRNA sequence features. tRNAviz implements a framework for determining consensus sequence features and can generate sequence feature distributions by isotypes, clades and anticodons, among other tRNA properties such as score. All visualizations are interactive and exportable. The web server is publicly available at http://trna.ucsc.edu/tRNAviz/.

## INTRODUCTION

In the era of thousands of genomes available for comparative study and bountiful sequencing-based molecular assays, our understanding of how full complements of tRNA genes are deployed and regulated is advancing. Transfer RNAs (tRNAs) are central to protein translation and are known to act as regulators of cell metabolism in multiple capacities (1–3). These functions are ultimately tied to the primary nucleotide sequence of the tRNA gene, which varies to a surprising degree between species across the domains of life, attesting to the continual evolution of this molecule (4–7). In many species, particularly multicellular eukaryotes, there are often multiple different versions of tRNAs with the same anticodon (isodecoders) that nominally carry out the same translation function (8,9). However, any difference in the regulation, processing and specific biological role(s) of the diversity of isodecoders is largely unexplored. The lifecycle and function of tRNAs is modulated by a huge array of proteins, many of which interact directly with the tRNA transcript, producing the most densely modified RNA in the cell (2,10–14). How somatic or germ-line tRNA mutations (e.g. single nucleotide polymorphisms) alter individual tRNA–protein interactions is not well defined for the vast majority of tRNAs and tRNA-processing proteins. For instance, a single unique Arg-TCT tRNA isodecoder is highly conserved throughout mammals which has a critical role in normal brain function (15), even though there are generally four to five other Arg-TCT isodecoders in these genomes. Many more cases of tRNAs with specialized function likely occur throughout the domains of life which could be identified for further study by examining exceptions to conservation patterns.

Within thousands of sequenced genomes and hundreds of thousands of tRNA genes (16–18), there is a unique opportunity to examine conservation patterns in any clade at the level of individual tRNA positions. However, the few tools designed for comparative genomic analysis of tRNAs lack query flexibility and sequence depth. Though tRNAdb (16), tRNADB-CE (17) and GtRNAdb (18) contain powerful search engines for filtering tRNAs by clade and isotype, further analysis is limited to downloading and exploring these sequences on one's own. A landmark comparative analysis of tRNAs (5), though comprehensive, was conducted early in the sequencing era with only 50 genomes. A thorough analysis of codon sparing in the three domains of life (19) took advantage of over a thousand additional genomes, but focused only on the anticodon (tRNA positions 34–36).

We developed tRNAviz to facilitate the study of conservation patterns of tRNA sequence features by any researcher with a web browser. tRNAviz can summarize consensus features, group and visualize sequence feature distributions across any combination of different phylogenetic clades, and probabilistically assess canonical or atypical nature of every position of user-provided tRNA sequences. All visualizations are downloadable as publication-quality figures, and over 150 000 tRNAs from over 1500 unique species are available for comparison and custom visualization.

## MATERIALS AND METHODS

tRNAviz was built with Python 3 and Django 2. Each module begins by including a form for choosing tRNAs based on the module's visualization(s). For most pages, upon form submission, the page refreshes with user input, then makes multiple queries to a public API, populating visualization panels with the returned results. User-input tRNAs and the pre-analyzed database of tRNAs are processed and annotated using tRNAscan-SE 2.0 (in preparation) which is based on the original tRNAscan-SE (20), and currently is available for online analysis (21) and source-code download. Briefly, the set of all raw tRNAscan-SE 2.0 predicted tRNAs decoding the standard twenty amino acids were used as the starting sequence set for tRNAviz, but the following additional filtering criteria were applied to remove tRNA-derived SINEs, pseudogenes, and other sequences that we judged unlikely to be involved in bacterial, archaeal or eukaryotic cytosolic ribosome translation: (i) tRNAs in phylum Chordata that do not belong to the high confidence gene set, (ii) tRNAs that are predicted as pseudogenes, (iii) genes with predicted truncation, (iv) fungal tRNA genes with scores below 50 bits, (v) all other tRNA genes with a score below 25 bits and (vi) predicted mitochondrial-origin tRNAs in nuclear mitochondrial DNA sequences (NUMTs; the vast majority of these genes were already filtered out as pseudogenes) (22). The mature tRNA sequences were then used to determine the consensus features and nucleotide distributions in tRNAviz. Phylogenetic lineages for each species were obtained by importing data from the National Center for Biotechnology Information (NCBI) using the taxize R library (23).

A consensus features framework was developed to simplify visual display of sequence patterns. We classified each position and base pair into a set of nucleotide ambiguity codes. For positions with high conservation of a particular nucleotide (e.g. purine), we summarized the distribution as a single consensus feature. Some positions were aggregated as base pairs before summarizing the distribution, yielding paired consensus features (e.g. Watson–Crick purine-pyrimidine base pair). Each possible base or base pair was required to exist in at least 5% of the tRNAs in question, in order to prevent rare features from making a disproportionate impact on the consensus determination. For example, to consider R57 as a consensus element, A57 and G57 each would need to be present in at least 5% of tRNAs, and their combined frequencies must reach the 90% threshold within each clade and isotype. The classification algorithm, using IUPAC nucleotide codes, first searched for zero ambiguity features (A, C, G, U, A:U, U:A, G:C, C:G, G:U, U:G), then partial ambiguity features (R, Y, W, S, M, K, R:Y, Y:R, S:S, W:W), then high ambiguity features (B, D, H, V, paired, mismatched). Using this system, consensus features are determined for all clades and species (Table 1).

**Table 1.** Summary of sources of tRNA data included in tRNAviz, Data Release 1.0

Domain	Species	tRNAs
Eukaryota	361	99 911
Bacteria	1035	63 002
Archaea	131	6343
Total	1527	169 256

All species names, taxonomy information and tRNA sequences in the current version of the website are accessible as text files from the ‘About’ page, and will be updated as new sequences are added.

In the Compare by sequence module, tRNAs are scored by first aligning the user-selected reference sequence(s) to the appropriate domain-specific tRNA covariance model (24) to assign standard Sprinzl position numbering (25). A new reference covariance model is built from this alignment. Then, each query tRNA group is aligned to this newly built reference model. For queries consisting of single tRNAs, the alignment shows position-specific bit values for the individual query sequence. For queries with multiple tRNAs, values displayed are instead averaged by position across all query sequences. All model building, alignment, and scoring steps are performed using Infernal (26). At last, all values for each position in query tRNAs are given as ‘penalty’ scores *p* = *q - r*, where the position-specific bit value derived from the reference covariance model (*r)* is subtracted from the score of the query sequence (*q*) at that same position. Thus, the highest penalty score (*p)* a query tRNA position can receive is zero if it is equal to the reference.

## RESULTS

### Summary visualizations

**Figure 1.**
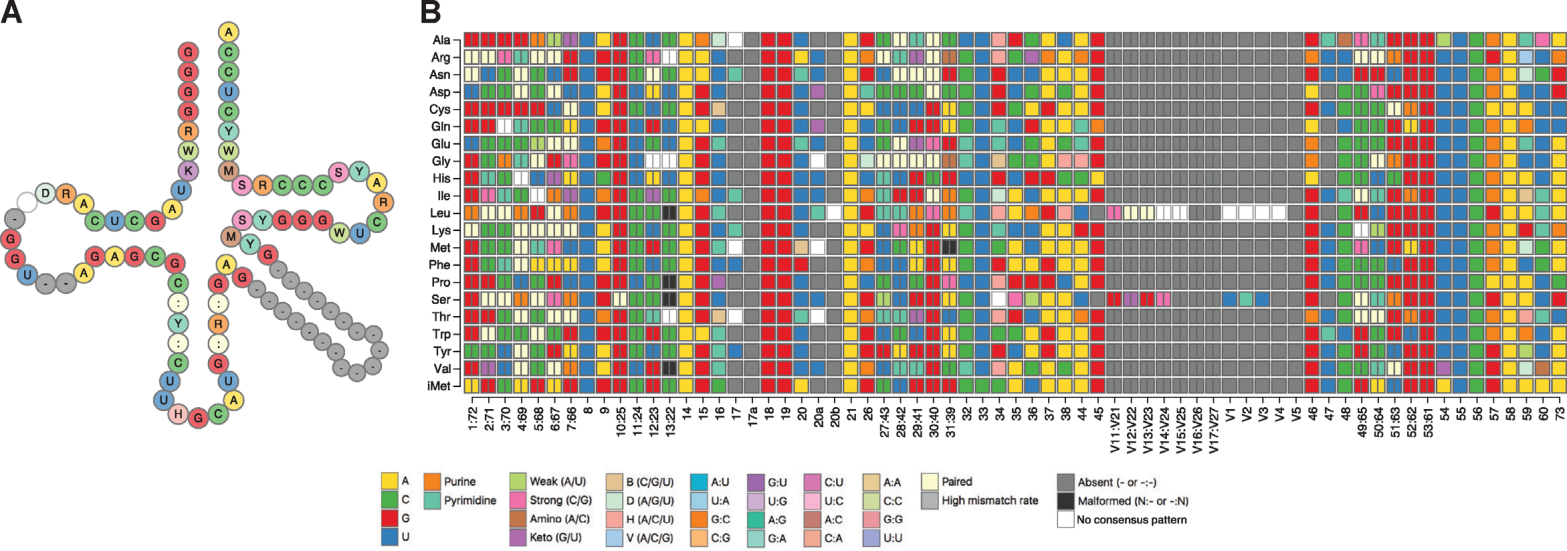
Consensus features are displayed on the Summary page. (**A**) A tRNA cloverleaf is visualized with the consensus sequence features of tRNA^Ala^ in primates. (**B**) A tilemap displays consensus isotype-specific features for primates. On the web server, mouseover of tiles (or cloverleaf positions) dynamically provides the base frequency distribution for the corresponding isotype and position. Colors correspond to nucleotide color legend.

The Summary page is the entry point into tRNAviz, giving users a quick, informative overview of conservation (nucleotide bias) for tRNAs from any single species, genus or clade (e.g. Mammalia) for a single isotype (e.g. Glycine), or calculated across all isotypes. There are multiple plots displayed on the Summary page: (i) a secondary structure ‘cloverleaf’ plot that displays pre-calculated consensus features (Figure 1A); and (ii) a ‘tilemap’ grid showing the breakdown of consensus features for every isotype (Figure 1B). Both plots are accompanied by a dynamic bar graph showing the distribution of nucleotides for any position in the cloverleaf or tilemap selected by user click or hover.

The Summary page also includes three tables that provide more contextual details of the information contained in the cloverleaf and tilemap visualizations. ‘Taxonomy summary’ shows counts of the number of tRNAs in the selected clade relative to all higher taxonomic clades (e.g. if the selected clade is ‘Fungi’, one can see that it contributes ∼25% of the total tRNAs in the Domain Eukaryota). This allows the user to assess how heavily weighted the selected clade is in calculations of domain-specific conserved features. The next table ‘Domain-specific consensus features’ highlights if the currently selected clade's tRNAs contain notable differences relative to the consensus of *all* the tRNAs in that domain. For each position, the clade's consensus feature is colored green in the table if it is an exact match against the domain's consensus feature, and colored red if it changes to a less specific or different consensus feature. Any change could hint at a possible evolutionary adaptation for one or more species in the selected clade. The ‘Anticodon counts’ table, sorted by isotype, provides details on the tRNA gene anticodon distribution. This information can potentially reveal changes in codon-anticodon decoding strategies, effected by a change in anticodon modifications patterns (6). Together, these tables illustrate potential sources of conservation bias and highlight species and new consensus features to investigate further.

### Compare tRNAs and visualize by clade or individual species

The Compare module aims to facilitate a deeper look at conservation patterns between different groups of species. Users select clade ‘groups’, which are user-specified combinations of clades, including domains all the way down to individual species. Clade groups are especially helpful for comparing sequence feature distributions among different species, regardless of ancestry. For instance, to examine tRNA evolution in Fungi with different life cycles, a user can choose to combine the high-level phyla Ascomycota (yeasts and sac fungi) with Basidiomycota (club fungi) in a single clade group, and compare their sequence feature distribution with Microsporidia (parasitic fungi). Clade groups can also be used to create arbitrary combinations. For instance, to see if there is a shared tRNA sequence signature among pathogenic bacteria, a user may choose to combine all known pathogenic bacteria in one clade group, opportunistically pathogenic bacteria in a second group and non-pathogenic bacteria in a third group. If there are unexpected patterns in distributions, subclades can be added to find the point in evolution where a new tRNA sequence pattern emerges.

The Clades page visualizes large full-page distributions for each clade group by user-specified position and isotype, and is useful for examining complex patterns such as aminoacyl–tRNA synthetase identity elements spanning specific positions of any desired isotype(s) and clades. Because the proportion of each nucleotide at each selected position is illustrated in bar graphs, exceptions to any patterns of interest can be quickly pinpointed. All positions, base pairs and tertiary interactions (e.g. position 10 with position 45) are supported, as are up to ten user-specified clade groups.

**Figure 2.**
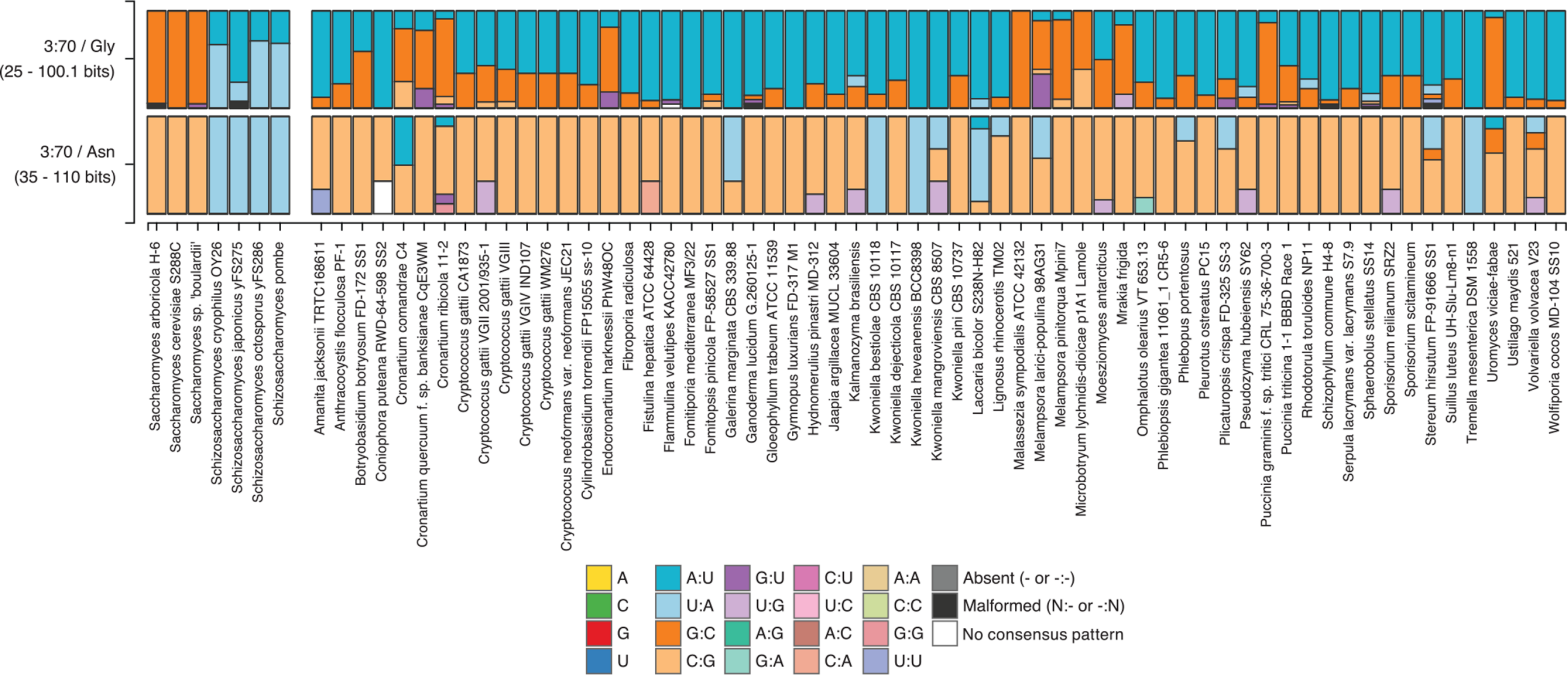
Compare tRNAs across species for focused isotypes/positions. Sequence feature distribution comparison shown between two clade groups (i) genera *Saccharomyces* and *Schizosaccharomyces*, and (ii) Basidiomycota phylum, at base pair 3:70 in tRNA^Gly^ and tRNA^Asn^. Bars represent a stacked histogram with colors corresponding to different nucleotide features. *Saccharomyces* have G:C or C:G exclusively at position 3:70 for these isotypes, whereas *Schizosaccharomyces* only use A:U or U:A at these positions. Basidiomycota species, by contrast, have a highly varied mix of base pair types at these positions. On the web server, mouseover of distribution bars reveals more detailed information about the specific feature.

The Species page visualizes distributions on a species by species basis, limiting each selection of tRNAs based on user-specified foci (Figure 2). Foci are a selection of tRNAs based on annotated features such as isotype, anticodon and tRNAscan-SE bit score (using a domain-specific, all-isotypes scoring model). For example, a user can elect to compare sequence feature distributions of high-scoring tRNA^Ala^ (e.g. ≥55 bits) versus low-scoring tRNA^Ala^ (e.g. <55 bits), then follow up by segregating by anticodon to find potential pseudogenes or tRNA-derived repetitive elements. These visualizations support any number of clade groups and foci, and clade groups are automatically separated within the generated figures.

### Explore taxonomy of tRNAviz species

The Taxonomy page serves as a reference page to view species included in tRNAviz. Starting with top-level domains, users can click into any clade to see what subclades are included within the database. The Taxonomy page provides two additional functions for each clade. First, upon clicking on the forked tree icon for a clade, a Newick-formatted tree is created containing only the clade and its subclades. The tree is then automatically uploaded to the Interactive Tree of Life (iTOL) website (27), which contains a dedicated suite of powerful visualization and tree manipulation tools. Second, for all clade levels except assembly and species, the arrow icon updates three tables on the lower portion of the Taxonomy page that provide context for the clade in question. Tables ‘tRNA counts by subclade’ and ‘Taxonomy tRNA statistics’ display tRNA counts and summary statistics for all direct subclades (direct children) of the selected clade. These tables provide information on how representative each subclade is of the entire clade. At last, the ‘tRNA scores by isotype’ table displays summary statistics for the clade with an isotype- and anticodon-specific focus.

### Analyzing user-specified or uploaded tRNA sequences

**Figure 3.**
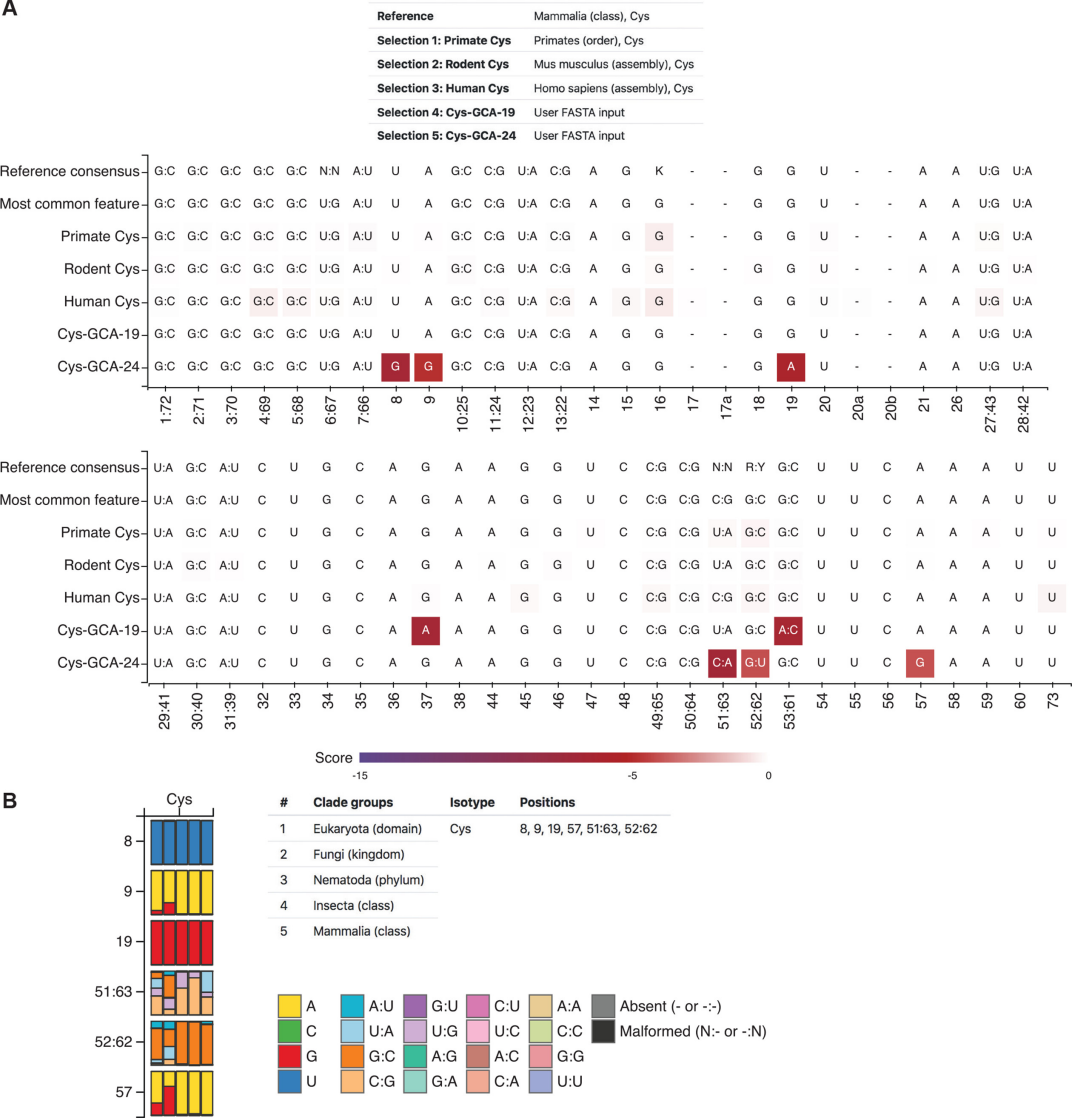
Bitchart and sequence feature distribution comparison for tRNA^Cys(GCA)^. (**A**) Position-specific sequence features with corresponding scoring scale for Cys-GCA-19 and Cys-GCA-24 in human are displayed in comparison with the consensus features in all human, mouse, primates and mammalian tRNA^Cys(GCA)^. The color scale of the features represents the conservation level with the reference. (**B**) Sequence feature distribution of tRNA^Cys(GCA)^ for six positions among five different clades is shown for comparison. The rows in the histogram represent the different positions of tRNA^Cys(GCA)^. The vertical bars (from left to right) within each row represent the five clades listed in the clade group table. The colors in the histogram represent the frequency of the corresponding sequence features (listed in the legend) at a particular position of a clade.

Although the analysis of pattern distributions described above is highly informative, users may wish to examine the sequence features of their own tRNAs. The Compare by sequence module allows users to align and score any particular tRNAs to a user-specified reference, and gives plots of position-specific scores in a bitchart (Figure 3A). Analysis requires (i) selection of a reference clade and optionally an isotype, and (ii) any number of query tRNAs groups. Query tRNA groups can either be pulled from the tRNAviz database via selection of clade and isotype, or submitted as one or more sequences in FASTA format. For its first row, a bitchart displays consensus features for the reference clade. The consensus features here are identical to those displayed via the Summary module. The second row shows the most common feature for each position. Each subsequent row displays a different query group, with the most common feature at each position superimposed onto a heatmap colored by the position's normalized score (see ‘Materials and Methods’ section). This visualization shows darker colors at positions that vary to a greater degree from the nucleotides in the reference sequences, focusing attention on the most important differences.

A common use case for this analysis is investigating why the tRNAscan-SE score for a user's sequence is very low: what feature(s) make that tRNA a ‘bad’ tRNA? The position(s) in the query sequence that cause the greatest penalty will be clearly be highlighted with this analysis. For instance, mutations may have occurred in the highly conserved A or B box internal promoters. Alternatively, a specific base pairing may have been lost which usually occurs in that isotype because it is required for proper aminoacylation (e.g. mutation of the G:U base pair identity element in alanine tRNAs at position 3:70).

Figure 3A illustrates this type of detailed positional analysis for two atypical human cysteine tRNAs, Cys-GCA-19 and Cys-GCA-24. The overall tRNAscan-SE bit scores do not indicate *why* these score lower than 20 other human cysteine tRNA genes, or what function(s) may be impaired. Figure 3A shows that Cys-GCA-19 has two mutations that lower the bit score (G > A at position 37, rare in mammals; and G > A at position 53, creating a non-Watson–Crick A:C base pair in the T-stem), pointing to features that might cause problems in processing or translation. Cys-GCA-24, however, has differences at six positions, five which are almost never seen in eukaryotic multicellular Cys tRNAs (U8 > G8, A9 > G9, G19 > A19, C62 > U62, A57 > G57)—a conclusion supported by examination of the frequency of each nucleotide in question by using the ‘Compare by Clade’ analysis for these positions (Figure 3B). These analyses suggest that Cys-GCA-24 is very likely to be a pseudogene based on many changes not observed in other tRNAs.

## DISCUSSION

In the past, assessing which sequence features are important for tRNA function has been difficult because of the limited number of biochemical studies of tRNA. If there are unusual features in a tRNA in a species and isotype that has never been studied experimentally, it can be extremely difficult for any researcher to determine if the differences represent evolution of a functional tRNA, or if the changes are likely to impair its proper function. Comparative sequence analysis offers a powerful tool to assess the most important elements of any DNA, RNA or protein for biological function by giving broader context, and has been central to guiding directed mutational studies for understanding essential sites of molecular interactions. Considering that there are millions of tRNAs sequenced across thousands of different species, the full power of this type of analysis has been out of reach for nearly all traditional tRNA researchers and those new to the field. We believe tRNAviz will become an essential tool to propel tRNA research forward by enabling flexible, facile analyses that lead to recognition of novel patterns, testable hypotheses and/or focused experiments for substantiating new tRNA biology.

## DATA AVAILABILITY

tRNAviz is an open source web application available to be used at http://trna.ucsc.edu/tRNAviz/. The source code can be obtained at GitHub (https://github.com/UCSC-LoweLab/tRNAviz-data and https://github.com/UCSC-LoweLab/tRNAviz).
